# Effects of acute hydration changes on cardiovascular magnetic resonance native T1 and T2 mapping

**DOI:** 10.1007/s10554-024-03291-9

**Published:** 2024-12-26

**Authors:** Katrine Aagaard Myhr, Emel Keceli, Joakim Bo Kunkel, Charlotte Burup Kristensen, Niels Vejlstrup, Lars Køber, Redi Pecini

**Affiliations:** 1 Department of Cardiology, The Heart Centre, Copenhagen University Hospital – Rigshospitalet, Inge Lehmanns Vej 7, Copenhagen, 2100 Denmark; 2 Cardiology, Department of Clinical Sciences, Lund University, Lund, Sweden

**Keywords:** Cardiovascular magnetic resonance, Native T1 mapping, T2 mapping, Hydration status

## Abstract

Changes in hydration status may affect myocardial native T1 and T2 values and influence the clinical interpretation. We aimed to assess the impact of acute preload augmentation on native T1 and T2. Cardiovascular magnetic resonance (CMR) native T1 and T2 mapping were performed twice on the same day in 20 healthy participants before and after an acute preload augmentation by a 2-liter intravenous infusion of isotonic sodium chloride (0.9%). Test-retest reproducibility was evaluated in 30 healthy participants with two consecutive CMR examinations on the same day. Sixteen participants were included in both substudies. In the 20 healthy participants undergoing acute preload augmentation (55% males, mean age (interquartile range [IQR]) 43 [29–51] years), native T1 increased with 17 ms (95% confidence interval [CI] 7 to 26; *p* = 0.001), T2 with 1.7 ms (95% CI 0.8 to 2.4; *p* < 0.001), and blood T1 with 46 ms (95% CI 28 to 65; *p* < 0.001). Test-retest variability in 30 healthy participants (47% males, median age 43 [28–52] years) showed 95% limits of agreement (LOA) of ± 26 ms for native T1, ± 2.1 ms for T2, and ± 57 ms for blood T1. In the 16 participants included in both substudies, the mean differences in changes post-infusion versus test-retest were 22 ms (95% CI 8 to 36; *p* = 0.01) for native T1, 1.9 ms (95% CI 0.9 to 2.9; *p* = 0.001) for T2, and 62 ms (95% CI 32 to 91; *p* < 0.001) for blood T1. Native T1 and T2 values increased following acute preload augmentation. However, the changes were within the 95% LOA of the test-retest reproducibility.

**Graphical Abstract:**

**Figure:**
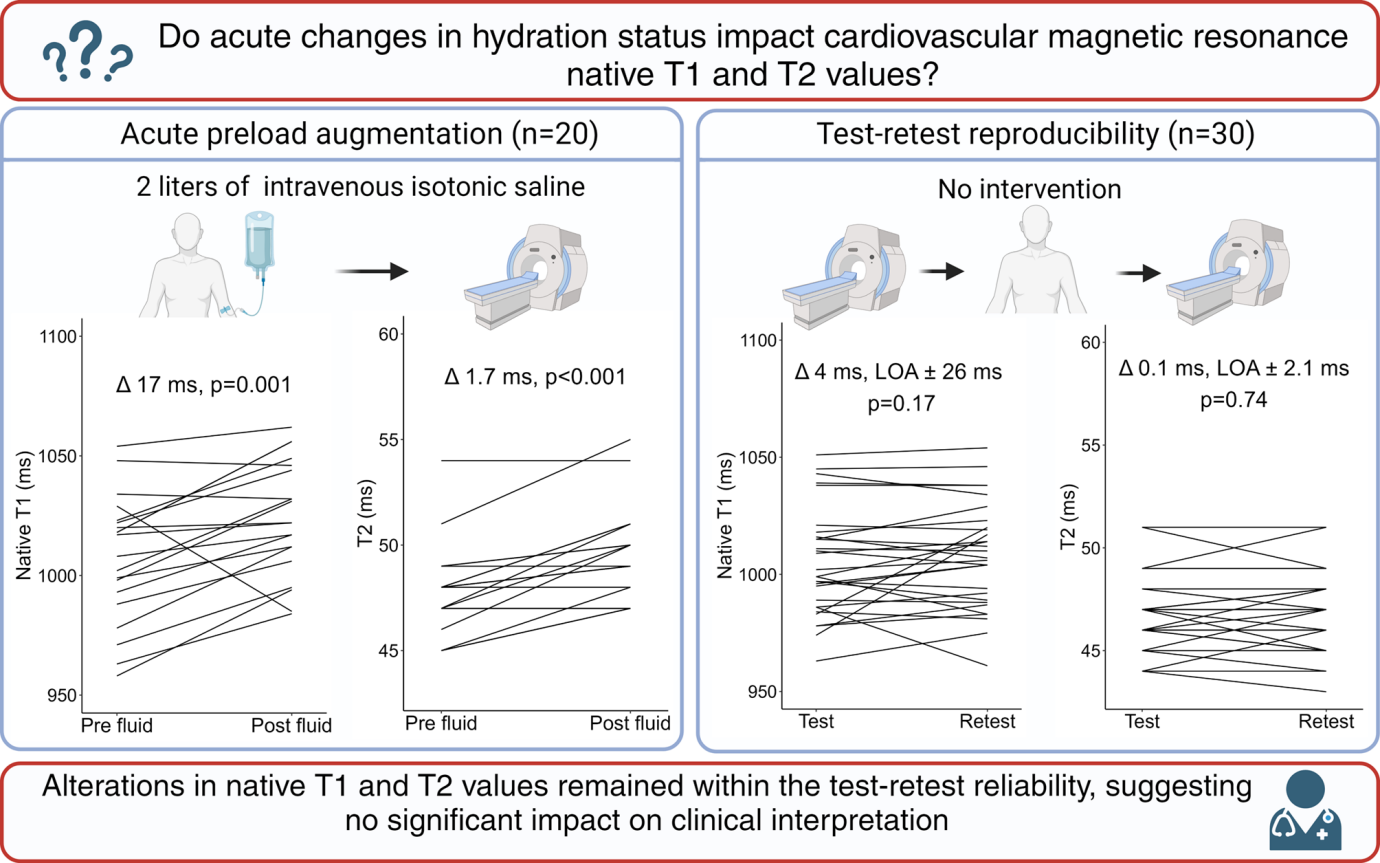
LOA, 95% limits of agreement. Created with BioRender

## Introduction

Cardiovascular magnetic resonance (CMR) native T1 and T2 mapping are non-invasive techniques for characterizing and quantifying myocardial tissue composition. Native T1 mapping measures the longitudinal relaxation time (T1) of myocardial protons and is frequently used as a surrogate marker of myocardial fibrosis. Further, native T1 has demonstrated clinical utility in the assessment of conditions such as myocarditis [1], Anderson-Fabry disease [2], amyloid disease [3], and iron overload [4]. Conversely, T2 values represent the transversal relaxation time of myocardial protons and are mainly affected by myocardial edema, such as in myocarditis [5, 6] or acute myocardial infarction [6, 7].

Native T1 and T2 relaxation times are both prolonged by increased interstitial water content [8, 9]. Consequently, changes in patients’ hydration status might affect the physical properties of the myocardium and thus significantly impact the clinical interpretability of native T1 and T2 values. If hydration status significantly influences native T1 and T2 values, it may be necessary to standardize patient preparation or adjust interpretation criteria in CMR assessments. However, the influence of hydration status on native T1 and T2 values remains understudied. Most existing research has centered on hemodialysis patients and has shown a reduction in native T1 and T2 values following dialysis [10, 11]. However, patients with renal failure commonly exhibit abnormalities in fluid volume distribution that do not reflect the physiological response of healthy individuals or those with fewer comorbidities [12].

The study aims to determine whether hydration status significantly affects the clinical interpretation of native T1 and T2 values. The primary aim was to investigate the impact of an acute change in hydration status on native T1 and T2 values in healthy participants. This was studied by subjecting patients to an intravenous infusion of 2 L of isotonic sodium chloride (0.9%), thus seeking to obtain an acute preload augmentation. Secondly, we sought to determine the test-retest reproducibility of native T1 and T2. Evaluating the test-retest reproducibility enabled us to determine whether any changes observed after preload augmentation exceeded normal measurement variability, thereby assessing their clinical significance.

## Methods

### Study population

Thirty-four healthy participants were recruited through online advertisement and included between October 2021 and September 2022 as part of a substudy in the clinical trial *Normal Variation of T1 Values with Cardiac Magnetic Resonance in Healthy Individuals (NATIVE)* (NCT05597657). The study participants were part of a larger population of healthy individuals previously described by our group [13]. Exclusion criteria were a history of any chronic disease, including cardiovascular, pulmonary, rheumatological, and renal disease. Further, ingesting antihypertensives, anticoagulants, lipid-lowering therapy, or other cardiovascular medications were exclusion criteria. CMR-specific exclusion criteria were claustrophobia, pregnancy, and non-compatible metallic implants. The study was conducted in accordance with the Declaration of Helsinki and approved by the Regional Ethics Committee for the Capital Region of Denmark (H-21025256). All participants signed written informed consent.

### Study design

**Fig. 1:**
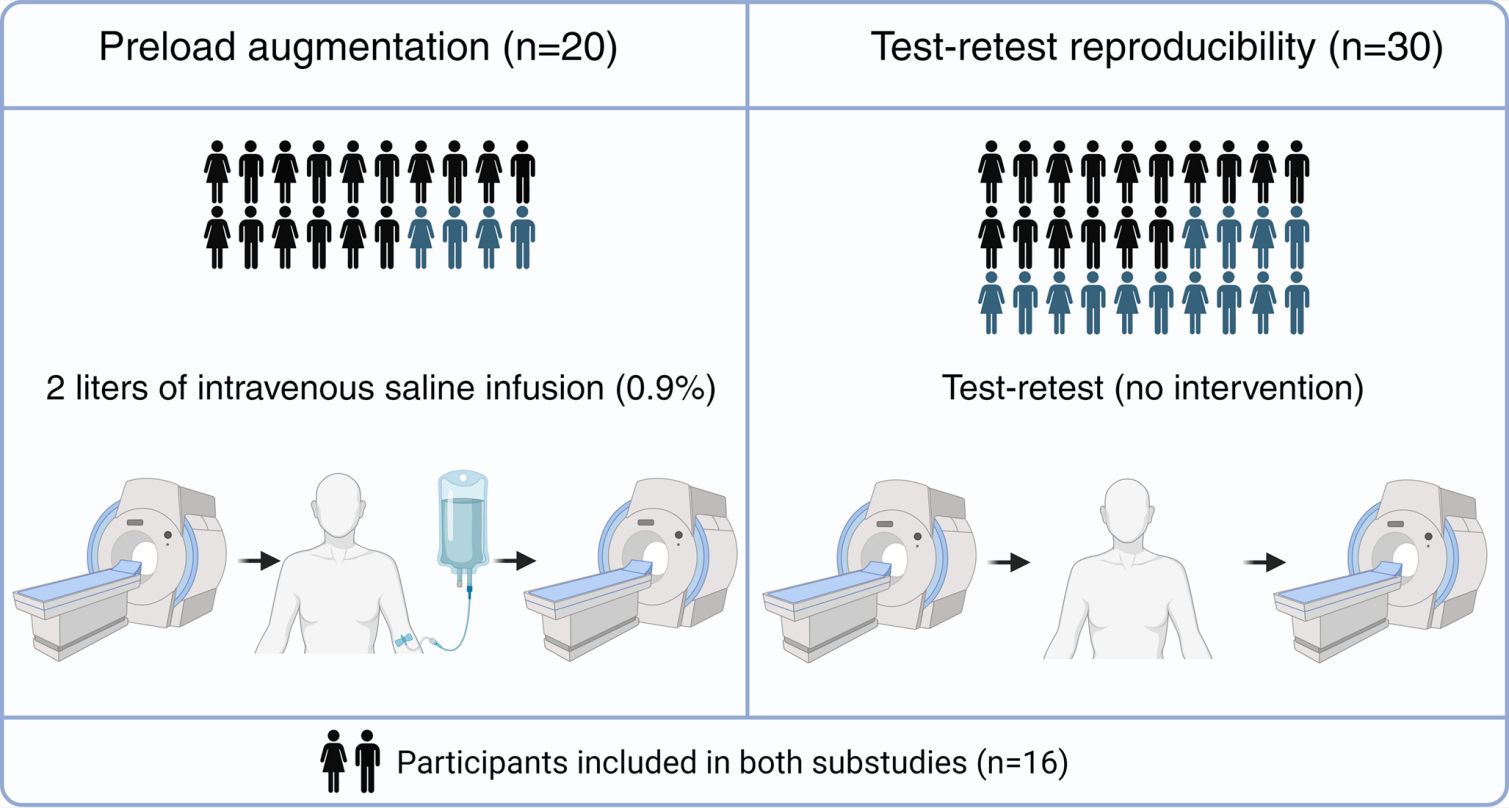
Study design. Created with BioRender

The study design is illustrated in Fig. 1. To evaluate the effects of preload augmentation on native T1 and T2, a subgroup of 20 participants underwent repeated CMR examinations on the same day. Immediately following the first scan, participants received an intravenous infusion of 2 L of isotonic sodium chloride (0.9%). A 2-liter infusion was chosen as we aimed to assess the impact of a clinically significant preload augmentation, and this amount had proven feasible and well-tolerated in a similar study by our group [14]. During the intravenous fluid administration, participants were placed stationary in a chair, and the infusion was supervised by a study team member. The participants were replaced in the scanner directly at the end of infusion. The infusion duration was 36 ± 9 min, corresponding to a rate of 59 ± 17 mL/min, and the time elapsed from the end of infusion to the acquisition of native T1 and T2 images was 30 ± 7 min. Blood pressure measurements were obtained in the supine position immediately following each CMR examination. Participants were not asked to refrain from eating or drinking before the baseline scan but did not consume any food or beverages between scans.

To assess the test-retest reproducibility of native T1 and T2, another subgroup of 30 participants underwent two consecutive scans on the same day. In between scans, participants were instructed to walk around relaxed for 3–5 min before being repositioned. Participants were invited to participate in both substudies; however, this was not mandatory. For those included in both substudies, all examinations were performed on the same day, and test-retest examinations were performed prior to the preload augmentation.

### CMR acquisition

CMR examinations were performed on a 1.5 Tesla Siemens MAGNETOM Aera Scanner with syngo MR software, version E11. The scanner and software were not modified during the study. Standard steady-state free precession (SSFP) cine images with retrospective gating were obtained during an expiratory breath hold to evaluate cardiac morphology and function. The imaging protocol included three long-axis views and a short-axis stack of the left ventricle (LV). A Look-Locker inversion recovery (MOLLI) sequence following a 5(3)3 scheme was used for native T1 mapping [15]. The parameters for this sequence were as follows: FOV 360 × 306.7 mm^2^, resolution 1.4 × 1.4 × 8 mm^3^, TR 280.6 ms, TE 1.12 ms, flip angle 35°. For T2 mapping, the imaging parameters were as follows: FOV 360 × 288.7 mm^2^, resolution 1.9 × 1.9 × 8 mm^3^, TR 193.3 ms, TE 1.06 ms, flip angle 70°. For both native T1 and T2 mapping, three short-axis slices of the LV were acquired: one mid-ventricular slice at the base of the papillary muscles; a second at the basal level, situated just beneath the LV outflow tract; and a third at the apical region, equidistant from the mid-ventricular slice, as previously described by our group [13].

### CMR analysis

CMR analyses were performed using Circle CVI42^®^ (version 5.13.5, Calgary, Canada) by a single experienced reader. All examinations were blinded and analyzed in a random order. LV volumes were determined using an AI-feature that automates the demarcation of endocardial and epicardial borders across all image slices and phases. Visual validation was subsequently performed. Motion-corrected MyoMaps were used for native T1 and T2 mapping analyses. Native T1 and T2 images were visually reviewed for significant artifacts. For both native T1 and T2 mapping analyses, endocardial and epicardial contours were drawn on all three short-axis slices. Care was taken to avoid the inclusion of the LV blood pool or surrounding structures. A conservative endo- and epicardial offset of 20% was chosen to avoid partial volume artifacts. Global native T1 and T2 values were subsequently generated.

### Statistics

**Fig. 2:**
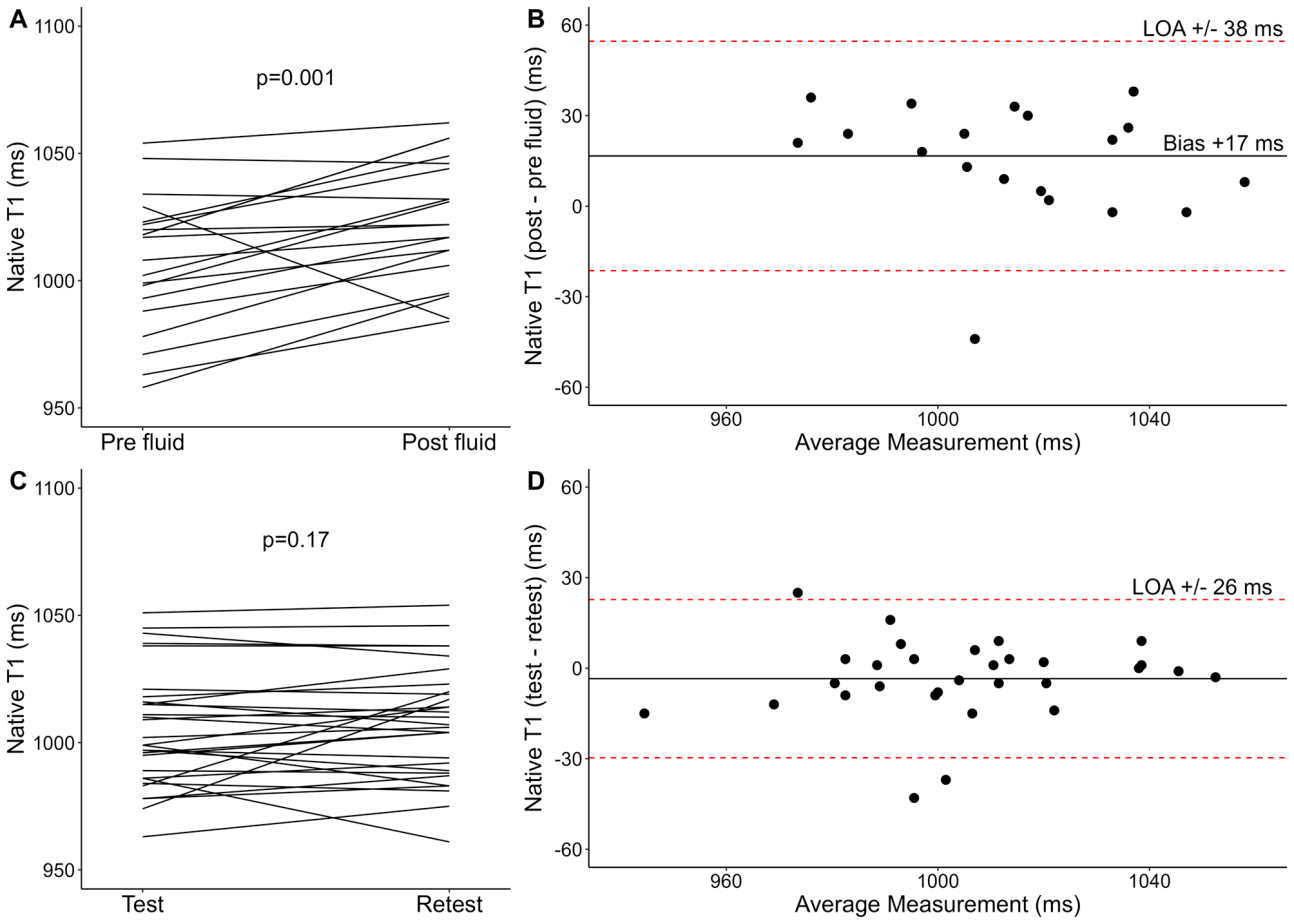
Alterations of native T1 following acute preload augmentation with an intravenous infusion of 2 L of isotonic sodium chloride (0.9%) (**A**–**B**) (*n* = 20) and test-retest reproducibility (**C**–**D**) (*n* = 30) presented as spaghetti plots (**A** and **C**) and Bland-Altman plots with mean bias (solid horizontal line) and 95% limits of agreement (LOA) (dashed, red lines) (**B** and **D**)

**Fig. 3:**
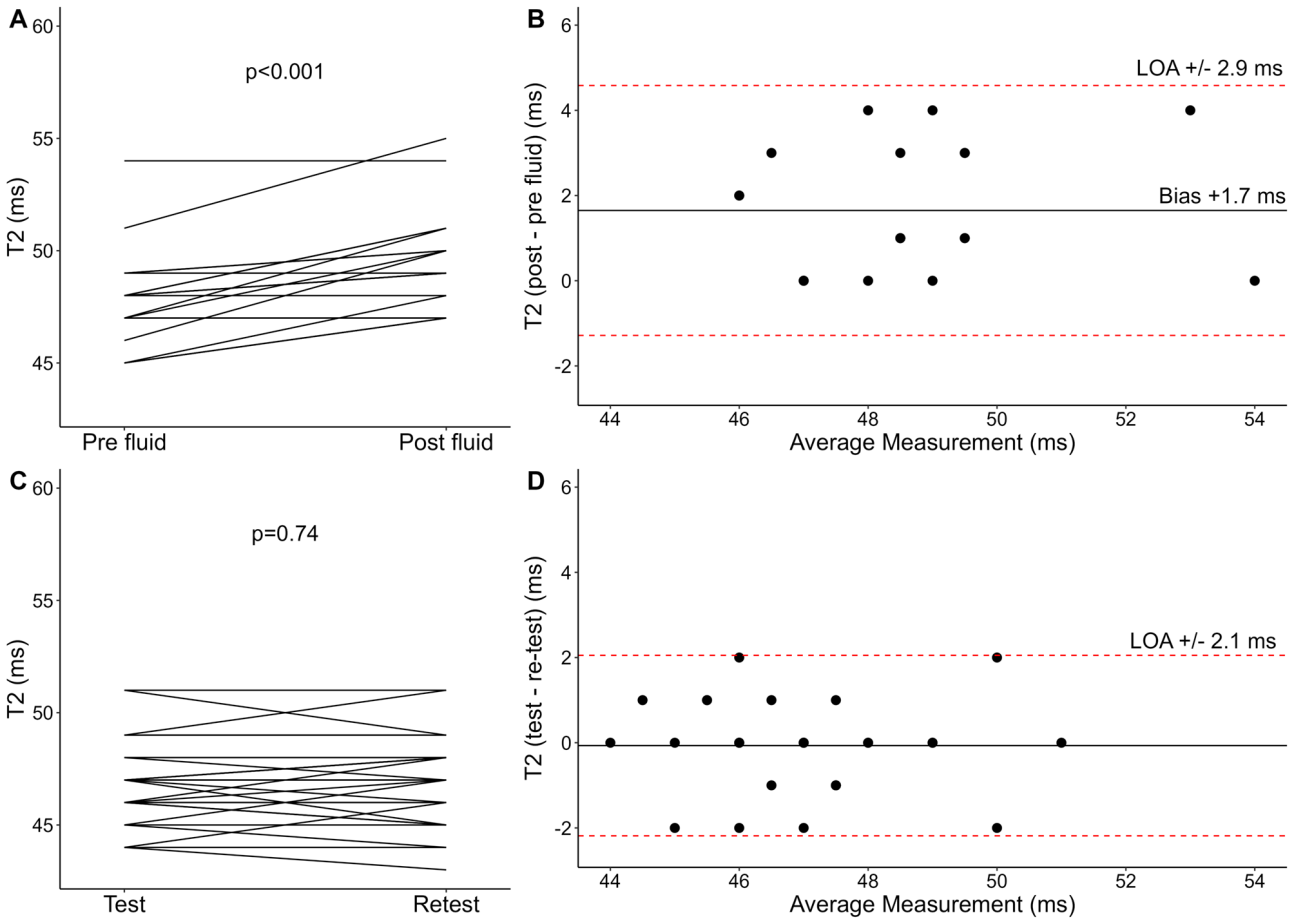
Alterations of T2 following acute preload augmentation with an intravenous infusion of 2 L of isotonic sodium chloride (0.9%) (**A**–**B**) (*n* = 20) and test-retest reproducibility (**C**–**D**) (*n* = 30) presented as spaghetti plots (**A** and **C**) and Bland-Altman plots with mean bias (solid horizontal line) and 95% limits of agreement (LOA) (dashed, red lines) (**B** and **D**)

**Fig. 4:**
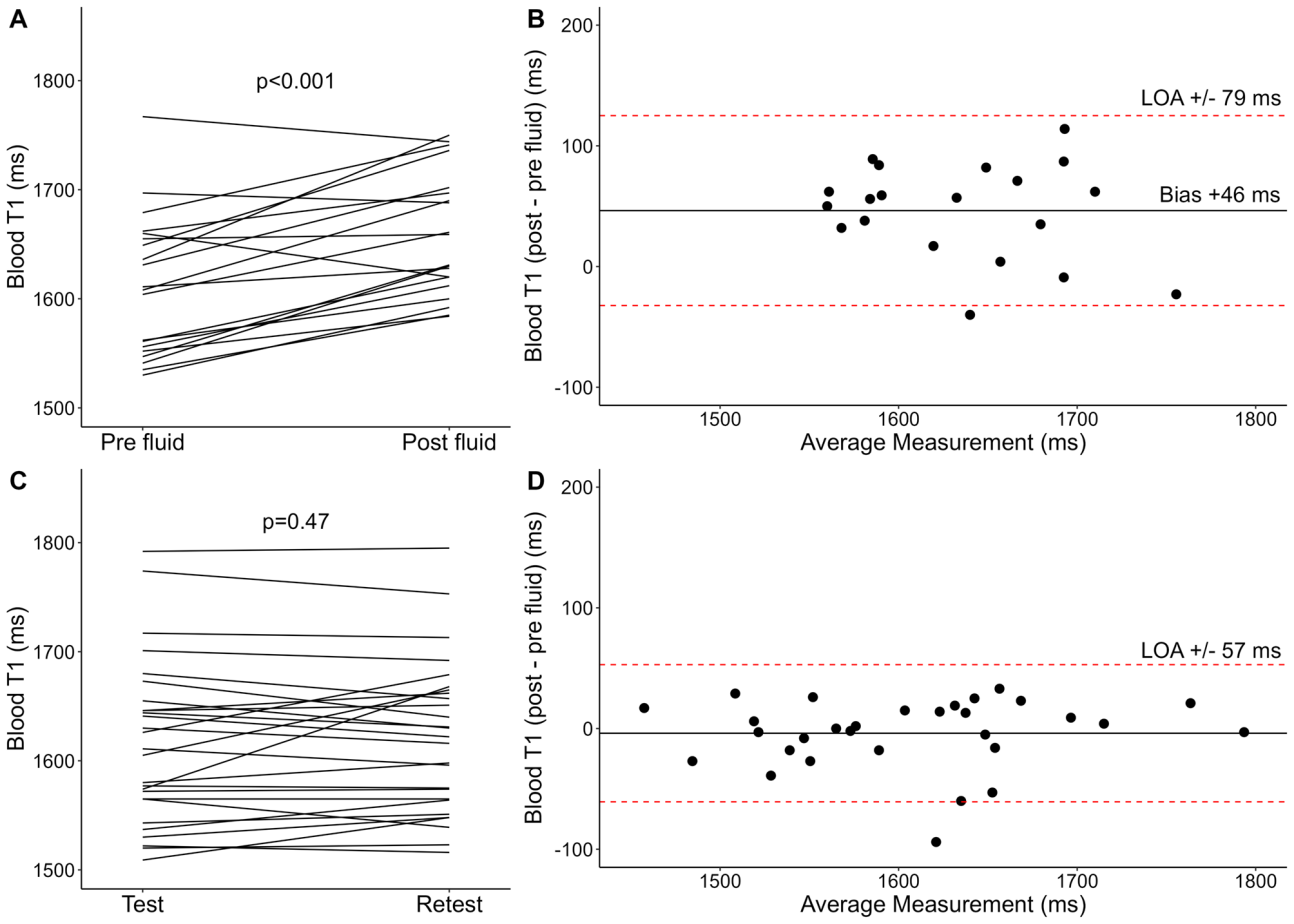
Alterations of blood T1 following acute preload augmentation with an intravenous infusion of 2 L of isotonic sodium chloride (0.9%) (**A**–**B**) (*n* = 20) and test-retest reproducibility (**C**–**D**) (*n* = 30) presented as spaghetti plots (**A** and **C**) and Bland-Altman plots with mean bias (solid horizontal line) and 95% limits of agreement (LOA) (dashed, red lines) (**B** and **D**)

Normally distributed data are presented as mean ± standard deviation, and non-normally distributed data as median (range) unless otherwise stated. Categorical data are presented as numbers (percentages). P-values ≤ 0.05 are considered statistically significant. Histograms and Q–Q plots assessed normality. Paired t-tests were applied for within-subject changes of native T1 (ΔT1), T2 (ΔT2), and blood T1 (Δblood T1). Bland-Altman plots were applied to visualize the 95% limits of agreement (LOA) and evaluate visual trends for changes following preload augmentation, as well as test-retest reproducibility. The within-subject coefficient of variation (CoV) by the root mean square method was calculated to evaluate the test-retest reproducibility of native T1 and T2. Pearson’s correlation coefficient (R) was used to assess the baseline correlation between native T1 and blood T1, the correlation between ΔT1 and ΔT2 following preload-augmentation, and as a post hoc test for the correlation between body surface area and native T1 and T2. R Statistical Software (v4.4.1; R Core Team 2024) was used for all statistical analyses and generation of Figs. 2, 3 and 4.

## Results

### Participant characteristics

Baseline demographic characteristics are presented in Table 1. Twenty healthy participants were included to evaluate the effect of preload augmentation on native T1 and T2. The median age was 43 (24–58) years, and 55% were male. All participants completed the 2-liter infusion of isotonic saline without notable side effects and were able to complete both CMR examinations. Additionally, 30 healthy participants were included to assess test-retest reproducibility of native T1 and T2. The median age was 43 (24–68) years, and 47% were male. Sixteen participants (median age 47 [24–58] years, 56% males) were included in both substudies.

**Table 1:** Baseline characteristics of the study populations that underwent acute preload augmentation (*n* = 20) and test-retest reproducibility examinations (*n* = 30), respectively

Characteristic	Preload augmentation (*n* = 20)	Test-retest reproducibility (*n* = 30)
Male sex, n (%)	11 (55%)	14 (47%)
Age, years	43 (29–51)	43 (28–52)
Weight, kg	75 ± 14	73 ± 14
Height, cm	176 ± 10	175 ± 9
BMI, kg/m^2^	24 ± 3	24 ± 3
BSA, m^2^	1.9 ± 0.2	1.9 ± 0.2

Values are n (%), mean ± standard deviation, or median (interquartile range)

BMI, body mass index; BSA, body surface area

### Preload augmentation

Native T1, T2, and blood T1 estimation was feasible in all participants. Native T1, T2, and blood T1 values increased following preload augmentation, as presented in Table 2; Figs. 2A and 3A, and Fig. 4A. Bland-Altman plots (Figs. 2B and 3B, and 4B) revealed no convincing trends. In post hoc analyses, changes in native T1 and T2 following preload augmentation did not correlate with body surface area (*R* = 0.30; 95% CI -0.20 to 0.65; *p* = 0.21; *R*= -0.05; 95% CI -0.48 to 0.40; *p* = 0.83; respectively).

**Table 2:** Alterations of physiological and cardiac magnetic resonance parameters following an intravenous infusion of 2 L of isotonic sodium chloride (0.9%) (*n* = 20) and test-retest reproducibility (*n* = 30)

Preload augmentation (*n* = 20)				
Parameter	Baseline	Preload augmentation	Δ (95% CI)	*P*-value
Diastolic blood pressure, mmHg	77 ± 13	80 ± 12	4 (0.2 to 8.6)	***0.042***
Systolic blood pressure, mmHg	118 ± 11	126 ± 14	8 (3.7 to 11.3	***< 0.001***
Heart rate, bpm	64 ± 11	63 ± 11	-1 (-4.0 to 1.2)	0.27
LVEDVi, ml/m^2^	86 ± 18	92 ± 15	6 (2.8 to 9.4)	***< 0.001***
LVESVi, ml/m^2^	33 ± 10	33 ± 11	1 (-0.9 to 2.0)	0.43
LVSVi, ml/m^2^	54 ± 10	59 ± 8	6 (2.6 to 8.5)	***< 0.001***
LVEF, %	63 ± 6	65 ± 6	2 (0.3 to 4.0)	***0.026***
Native T1, ms	1007 ± 26	1024 ± 24	17 (8 to 26)	***0.001***
T2, ms	48 ± 2	49 ± 2	2 (1 to 2)	***< 0.001***
Blood T1, ms	1612 ± 64	1658 ± 56	46 (28 to 65)	***< 0.001***
**Test-retest reproducibility (n = 30)**				
**Parameter**	**Test**	**Re-test**	**Δ (95% CI)**	**P-value**
Heart rate, bpm	66 ± 10	64 ± 9	-3 (-4 to -1)	***< 0.001***
LVEDVi, ml/m^2^	87 ± 15	87 ± 12	-1 (-4 to 1)	0.24
LVESVi, ml/m^2^	32 ± 7	32 ± 7	-0.1 (1 to -1)	0.83
LVSVi, ml/m^2^	55 ± 10	55 ± 10	-1 (-4 to 1)	0.31
LVEF, %	63 ± 4	63 ± 4	-0.4 (-2 to 1)	0.61
Native T1, ms	1002 ± 26	1006 ± 24	4 (-2 to 8)	0.17
T2, ms	47 ± 2	47 ± 2	0.1 (-0.3 to 0.5)	0.74
Blood T1, ms	1603 ± 82	1607 ± 79	4 (-7 to 15)	0.48

Values are n (%) or mean ± standard deviation. Significant p-values are in bold italics

LVEDVi, left ventricular end-diastolic volume index; LVEF, left ventricular ejection fraction; LVESVi, left ventricular end-systolic volume index; LVSVi, left ventricular stroke volume index

**Fig. 5:**
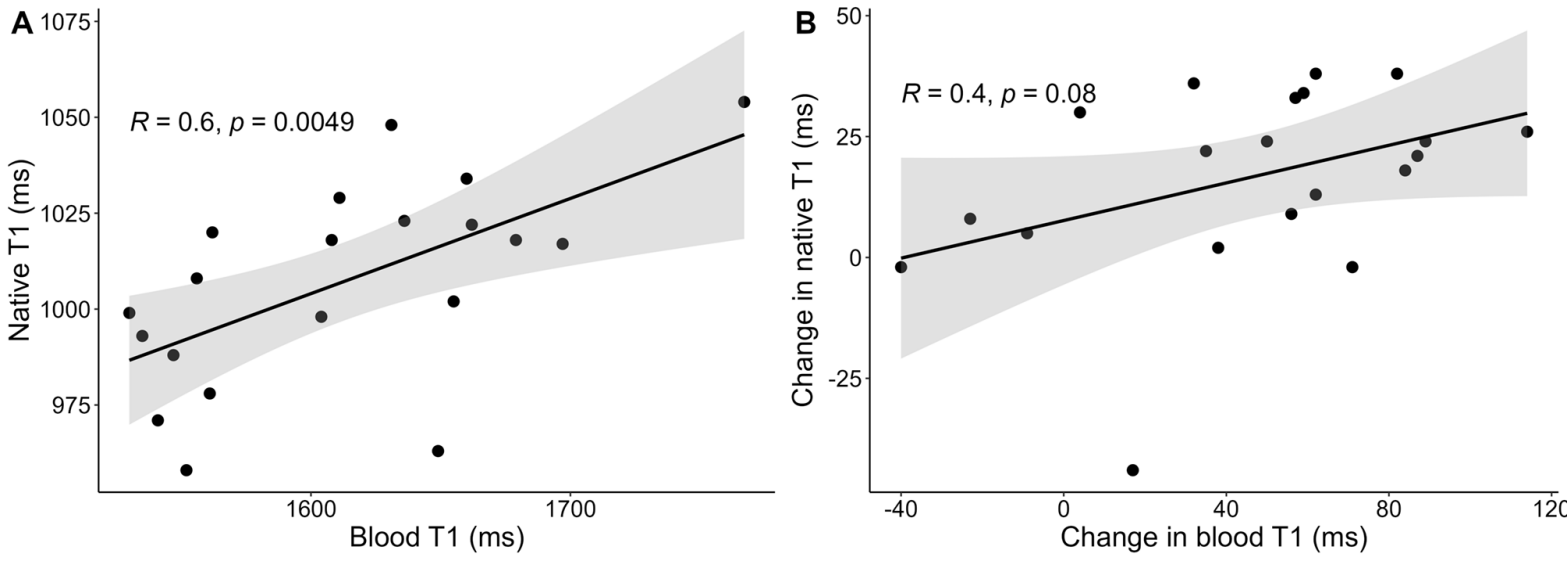
Correlation between native T1 and blood T1 at baseline (**A**) as well as between changes in native T1 and blood T1 (**B**) following preload augmentation with an intravenous infusion of 2 L of isotonic sodium chloride (0.9%). R, Pearson’s correlation coefficient

Native T1 and blood T1 correlated at baseline (*R* = 0.6; 95% CI 0.22 to 0.83; *p* = 0.005), as illustrated in Fig. 5A. The correlation between alterations in native T1 and blood T1 following preload augmentation did not reach statistical significance (*R* = 0.40; 95% CI -0.05 to 0.72; *p* = 0.08).

Alterations of physiological and CMR parameters following preload augmentation and test-retest exams are presented in Table 2. Diastolic and systolic blood pressure, LV end-diastolic volume index, LV stroke volume index, and left ventricular ejection fraction (LVEF), all increased following preload augmentation (*p* < 0.05). LV volumes and function remained unaltered during test-retest exams; however, heart rate decreased by 3 beats per minute at retest exams (95% CI -4 to -1; *p* < 0.001).

### Test-retest reproducibility

Thirty healthy participants underwent repeated CMR to assess the test-retest reproducibility of native T1, T2, and blood T1. The results are illustrated in Table 2; Figs. 2C–D and 3C–D, and Fig. 4C–D. The 95% LOA was ± 26 ms for native T1, ± 2.1 ms for T2, and ± 57 ms for blood T1. The within-subject CoV was 1.0% for native T1 and 1.6% for T2.

### Direct comparison of preload augmentation and test-retest reproducibility

A direct comparison of changes following preload augmentation and changes following test-retest was performed for the 16 participants included in both substudies. Preload augmentation resulted in an average increase of 22 ms in native T1 (95% CI 8 to 36; *p* = 0.005), an average increase of 1.9 ms in T2 (95% CI 0.9 to 2.9; *p* = 0.001), and an average increase of 62 ms in blood T1 (95% CI 32 to 91; *p* < 0.001) compared with the test-retest examination.

## Discussion

In this CMR study of healthy individuals, we found that myocardial native T1 and T2 increased in response to a 2-liter intravenous saline infusion compared with test-retest measurements. However, the observed average increments in native T1 and T2 were within the 95% LOA of test-retest reliability. These findings suggest that acute changes in hydration status, as applied in this study, do not significantly affect the clinical interpretation of native T1 and T2 values. With this study, we believe we are the first to describe the alterations of native T1 and T2 following preload augmentation through intravenous saline infusion.

Previous studies examining the effect of hydration status on native T1 and T2 have mainly concentrated on hemodialysis patients. This focus may be attributed to several factors. First, hemodialysis patients represent a multimorbid patient group in whom the evaluation of fibrosis and/or edema with native T1 and T2 mapping might be of clinical relevance but in which significant alterations in hydration status due to hemodialysis might affect the interpretability. Second, this patient group provides a practical model to study the effects of acute hydration changes non-invasively. Two studies with native T1 mapping before and after hemodialysis in 25 and 26 patients found a decline in both native T1 and T2 following dialysis [10, 11], which is in line with our study. Other cross-sectional studies have examined the correlation between native T1 and hydration status by using bioimpedance measurements. Antlanger et al. [16] reported higher native T1 values in fluid-overloaded hemodialysis patients compared with healthy controls. In a study by Donà et al. [17] involving 285 patients referred for CMR for various conditions, overhydration was significantly associated with elevated native T1. Another large outcome study by Treiber et al. [18] of 2047 patients referred for CMR showed that native T1 and T2 were elevated in patients with volume expansion as calculated from Hakim’s formula, which incorporates hematocrit, weight, and sex.

To our knowledge, the study by Luetkens et al. [19] is the closest in design to our study. They included 12 healthy participants who underwent a baseline scan, a dehydration scan after 12 h without food or beverage intake, and a hydration scan 15 min after drinking at least 1.5 L of water. They reported a decrease in native T1 from 987 ms at baseline to 968 ms after dehydration, followed by an increase to 986 ms after rehydration. Similarly, T2 decreased from 52.9 ms to 51.5 ms after dehydration and increased to 52.2 ms following rehydration. As in our study, the CMR protocol included a 5(3)3 MOLLI scheme on a 1.5 Tesla scanner; however, the scanner was a Philips, contrary to Siemens utilized in our study, and a single midventricular slice was acquired as opposed to three slices. Even though the absolute values are not directly comparable to our study due to these discrepancies in methodology, the dehydration-hydration changes in native T1 seem comparable to our findings. An important difference between the studies lies in the method of hydration. We opted for an intravenous saline infusion with patient monitoring between scans, thus ensuring a highly controlled setting. In comparison, oral fluid uptake is presumably more exposed to inter-individual variations in uptake time and amount. Prior to performing the current study, our group tested a similar setting with rapid oral intake of 2 L of tap water (unpublished data). We found that a large amount of water was still contained in the stomach 30 min following intake and that this water produced significant artifacts on the T1 mapping images. Therefore, we continued with an intravenous setup. However, a possible strength in the study by Luetkens et al. [17] is the assessment of the difference in native T1 and T2 at baseline compared with subsequent dehydration/fasting. In a recent study, our group reported significant effects of food intake on cardiovascular hemodynamics on CMR, mainly by increasing heart rate and LV contractility [20]. However, we chose not to require fasting for participants, aiming to mirror typical clinical settings where patients are not routinely asked to fast before a CMR examination. We believe this approach ensures a more natural hydration state, providing a more accurate representation of how acute hydration changes in a general population could affect native T1 and T2 values, thereby enhancing the generalizability of the study. More importantly, patients did not consume any food or beverages between scans, which could have introduced unwanted variability.

The main purpose of this study was to pragmatically assess any variations of native T1 and T2 caused by changes in hydration status, regardless of underlying mechanisms. We cannot determine how the infused saline was distributed among the different fluid compartments and, therefore, by which mechanism T1 and T2 values were affected. However, since LV volumes increased after the infusion, some saline remained in the vascular compartment during the post-infusion CMR exam. This would decrease hematocrit levels with a corresponding increase in blood T1, as these two are correlated [21]. In our study, blood T1 increased significantly by 46 ms compared with a non-significant change during test-retest exams. The rise in blood T1 may have increased T1 values due to partial volume effects, either from the LV blood pool or intravascular compartment. To explore this, we analyzed the correlation between ΔT1 and Δblood T1, which was insignificant. Even though there was a risk of Type II error, this suggests that the increase in native T1 may be at least partly attributed to enhanced interstitial and/or intracellular myocardial water content rather than solely to blood T1 alterations. This is supported by the findings from Rankin et al. [10], who similarly observed no correlation between the decrease in native T1 post-hemodialysis and decreases in blood T1 and hematocrit levels.

This study may have several clinical implications. Even though native T1 and T2 increased significantly following a 2-liter acute preload augmentation, the average increments did not exceed the test-retest uncertainty. Hence, our results indicate that such changes in hydration status do not significantly affect clinical interpretability. This may be valuable when assessing follow-up patients with unknown hydration status. Further, the observed alterations in this study were minor compared to the significant alterations seen in acute myocardial infarction or myocarditis, where native T1 and T2 values compared with healthy controls are elevated by ~ 50–80 ms [1, 5, 22, 23] and ~ 6–9 ms [5, 6, 22], respectively. As this study involved healthy individuals, other factors affecting the distribution of fluids may dominate in different patient groups, such as electrolyte derangements, infections, cardiac, renal, and hepatic disease. Therefore, future research should focus on exploring the impact of hydration status on native T1 and T2 values across different patient populations, such as patients with heart failure, valvular heart disease, and cardiomyopathies.

### Limitations

This study is limited by the small sample size. Consequently, we could not adjust for potential confounders, such as blood T1 and body size. Besides demonstrating that the preload augmentation was mirrored in LV volumes and function alterations as compared with test-retest examinations, we did not objectively assess hydration status before and after the intravenous saline infusion, such as measuring hematocrit levels, other blood compounds, bioimpedance analysis, or urine analysis. However, we opted for a pragmatic approach where the primary aim was to assess clinically relevant changes in native T1 and T2 rather than to determine fluid compartmentalization and its impact on these values. Further, all participants were administered the same amount of intravenous saline irrespective of body size. This likely resulted in varying relative increases in preload and potentially different water distribution to the myocardium. Adjusting the infused volume according to body size might have provided more accurate estimates of the absolute increase in native T1 and T2 for a given relative infused volume. However, the increases in native T1 and T2 did not show a correlation to body surface area. While this could be attributed to the small sample size, which included only adults with limited body size variation, it may also suggest that adjusting the infused volume based on body size might not significantly impact these values. Also, various other factors influencing the distribution would still introduce uncertainties to these estimates, and we believe that a 2-liter infusion constitutes a substantial preload augmentation regardless of body size. Lastly, heart rate decreased by 3 beats per minute during the test-retest reproducibility exams, likely because participants were more relaxed. Although native T1 may be slightly heart rate dependent, we do not believe this small difference has significantly affected the results.

## Conclusion

Myocardial T1 and T2 values increased in healthy individuals in response to acute preload augmentation obtained from a 2-liter intravenous saline infusion. However, the average increments in native T1 and T2 values fell within the bounds of test-retest reliability. These findings suggest that routine adjustment for hydration status may not be necessary when interpreting native T1 and T2 values in routine clinical practice.

## Data Availability

No datasets were generated or analysed during the current study.
